# The Development of New Primer Sets for the Amplification and Sequencing of the Envelope Gene of All Dengue Virus Serotypes

**DOI:** 10.3390/microorganisms12061092

**Published:** 2024-05-28

**Authors:** Stefania Fraenkel, Takeshi Nabeshima, Dalouny Xayavong, Thi Thanh Ngan Nguyen, Qiang Xu, Merveille Kapandji, Kano Yamao, Jean Claude Balingit, Basu Dev Pandey, Kouichi Morita, Futoshi Hasebe, Mya Myat Ngwe Tun, Yuki Takamatsu

**Affiliations:** 1 Department of Virology, Institute of Tropical Medicine (ITM-NU), Nagasaki University, Nagasaki 852-8523, Japan; stefifraenkel@gmail.com (S.F.); mtmikami@tm.nagasaki-u.ac.jp (T.N.); dlnxyv93@gmail.com (D.X.); thanhngan0605@gmail.com (T.T.N.N.); xq101325@126.com (Q.X.); mckkapandji@gmail.com (M.K.); bb25120044@ms.nagasaki-u.ac.jp (K.Y.); jcpbalingit@gmail.com (J.C.B.); drbasupandey@gmail.com (B.D.P.); moritak@nagasaki-u.ac.jp (K.M.); rainbow@nagasaki-u.ac.jp (F.H.); myamyat@tm.nagasaki-u.ac.jp (M.M.N.T.); 2 School of Tropical Medicine and Global Health (TMGH), Nagasaki University, Nagasaki 852-8523, Japan; 3 Kenya Research Station, Institute of Tropical Medicine (ITM-NU), Nagasaki University, Nagasaki 852-8523, Japan; 4 Graduate School of Biomedical Sciences, Nagasaki University, Nagasaki 852-8523, Japan; 5 Dental School, Nagasaki University, Nagasaki 852-8588, Japan; 6 DEJIMA Infectious Disease Research Alliance, Nagasaki University, Nagasaki 852-8523, Japan; 7 Center for Vaccines and Therapeutic Antibodies for Emerging Infectious Diseases, Shimane University, Izumo 690-8504, Japan; 8 Vietnam Research Station, Institute of Tropical Medicine (ITM-NU), Nagasaki University, Nagasaki 852-8523, Japan

**Keywords:** DENV, RT-PCR, sequencing, serotype-specific

## Abstract

Dengue virus (DENV) poses a significant threat to global health, infecting approximately 390 million people annually. This virus comprises four serotypes capable of causing severe disease. Genetic analyses are crucial for understanding the epidemiology, evolution, and spread of DENV. Although previous studies have focused on the envelope protein-coding (E) gene, only a few primers can efficiently detect and amplify the viral genes from multiple endemic countries simultaneously. In this study, we designed degenerate primer pairs for each DENV serotype to amplify and sequence the entire E gene, using globally representative sequences for each serotype. These primers were validated using DENV isolates from various Asian countries and demonstrated broad-spectrum detection capabilities and high-quality sequences. The primers provide effective tools for genetic analysis in the regions affected by dengue, aiding strain identification and epidemiological studies during outbreaks.

## 1. Introduction

Dengue virus (DENV) is a single-stranded positive-sense RNA virus belonging to the *Flaviviridae* family that causes dengue fever, a mosquito-borne viral disease that infects an estimated 390 million people annually worldwide [1,2]. Most dengue fever cases are asymptomatic, but some individuals experience mild symptoms, such as fever, myalgia, and rash. Among these cases, 5–20% progress to severe dengue with clinical manifestations that include plasma leakage and fluid accumulation leading to shock, respiratory distress, severe bleeding, and severe organ impairment [3]. Severe dengue can be life-threatening and requires immediate medical support [4,5].

There are four distinct DENV serotypes (DENV-1–DENV-4), all capable of causing severe dengue [4]. Previous studies have indicated that heterotypic secondary dengue infections pose an increased risk for severe forms of dengue [6,7]. Moreover, closely related strains are clustered into genotypes, with five to seven genotypes defined for each DENV serotype. The genetic differences between serotypes and genotypes are crucial factors that determine viral fitness, pathogenicity, and epidemic potential [8,9].

Dengue fever is prevalent in both the tropical and subtropical regions. However, there has been a gradual shift, with the disease being reported in more temperate regions. For example, autochthonous cases were reported in Croatia and France [10,11]. Not only has its geographical distribution expanded but its incidence has also increased 30-fold from 1960 to 2010, and this continues to increase. This expansion has been attributed to climate change, urbanisation, globalisation, and the spread of *Aedes* mosquitoes, which are disease vectors. 

The regions most affected by dengue are Southeast Asia, the Western Pacific, and the Americas. Asia alone accounts for 70% of the global dengue burden, as reported by the World Health Organization in 2023 [12]. All four DENV serotypes circulate in most Southeast Asian areas and have caused several epidemics. The occurrence of large outbreaks can be attributed to various factors, such as the introduction of a new viral lineage, a shift in the dominant viral strain, climatic factors, environmental conditions, vector–host interactions, vector competence, and host susceptibility [13,14].

From 2022 to 2023, several Asian countries have reported a significant increase in dengue incidence compared with previous years. Longer seasonal dengue outbreaks and geographic expansion have also been observed in previous reports from the WHO Regional Office for Southeast Asia and the Western Pacific Region [15,16]. Since there is often a link between an increase in dengue incidence and the emergence of a specific DENV lineage, it is crucial to study the genetic diversity of DENV. Molecular phylogenetic analyses and studying the viral genome are essential for tracing the history of DENV epidemics over time and across various locations. Through DENV genome analysis, valuable insights can be gained into the origin, evolution, and spread of the virus, thereby enhancing the ability to track and respond effectively to dengue outbreaks [17,18].

Over the years, DENV genetic diversity has been studied primarily based on the envelope (E) protein-coding gene, the most antigenically essential viral protein. Approximately 70% of this gene is conserved across all DENV serotypes, making it possible to determine the serotype of the strain by sequencing this gene [4,19]. However, owing to the genetic variability within the DENV population, it is advantageous to design primers specific to particular genotypes or lineages of the virus to ensure the accurate amplification and sequencing of the target gene. Several reports have described primer sets designed to detect the viral genome in specific endemic countries without testing them using samples from other regions [20,21]. To our knowledge, the only primers capable of efficiently detecting and amplifying the entire E gene from multiple endemic countries simultaneously were developed by the Centers for Disease Control and Prevention (CDC) and tested only on samples from the Americas [22]. This study established a molecular methodology for amplifying and sequencing the E gene of DENV strains from Southeast Asia. 

## 2. Materials and Methods

### 2.1. Ethics Statement

This study was approved by the Research Ethics Committee of Nagasaki University Institute of Tropical Medicine, Japan (230209288, 27 February 2023).

### 2.2. Design of Oligonucleotides

**Figure 1:**
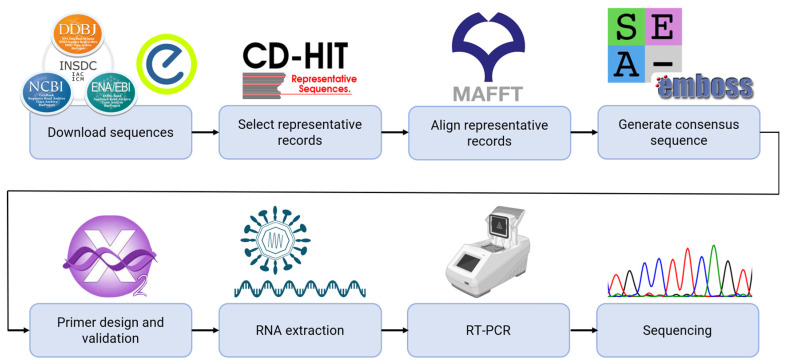
Schematic of the methodology used to design primers for each dengue virus (DENV) serotype. All available genomic RNA sequences of DENV were downloaded from the International Nucleotide Sequence Database Collaboration. Subsequently, 100–200 representative records for each serotype were selected using CD-HIT-EST (v4.8.1). These records were then aligned using MAFFT (v7.515), and the aligned sequences were further analysed using SeaView (v5.0.5) and EMBOSS (v6.6.0) to generate a consensus sequence for each serotype. To amplify the complete E gene of each serotype, the terminal region of the prM gene and the beginning of the NS1 gene were analysed to design the primers. The primers were designed using Primer3 (v2.6.1) and subjected to validation using AmplifX2 (v2.1.1). Following the same procedure, primers spanning the entire E gene were designed for each serotype for the Sanger sequencing. The DENV envelope gene was amplified through an RT-PCR assay, followed by Sanger sequencing using our newly designed primers.

New primers were designed to develop an RT-PCR assay to amplify the entire DENV-E gene. To achieve this goal, all available genomic RNA sequences of DENV were downloaded from the International Nucleotide Sequence Database Collaboration. Subsequently, 100–200 representative records of each serotype were selected using CD-HIT-EST (v4.8.1) [23]. These records were aligned using MAFFT (v7.515) [24], and the aligned sequences were further analysed using the SeaView multiplatform graphical user interface (GUI) for molecular phylogeny (v5.0.5) [25] and EMBOSS (v6.6.0) [26] to generate a consensus sequence for each serotype. To amplify the complete E gene of each serotype, the terminal region of the prM gene and the beginning of the Nonstructural Protein 1 (NS1) gene were analysed to design the primers. The following criteria were used when designing the primers: (1) maximum homology within each serotype and (2) nonhomology to other regions of the DENV genome. Although the maximum homology was sought, there was a significant variation within each serotype. Therefore, degeneracy was introduced into the primers to accommodate the variability revealed based on the alignment. The primers were designed using Primer3 (v2.6.1) [27], and they were subjected to validation using AmplifX2 (v2.1.1) (Figure 1). Following the same procedure, primers spanning the entire E gene were designed for each serotype for the Sanger sequencing.

### 2.3. Viral RNA Extraction

Viral RNA was extracted using the QIAamp^®^ Viral RNA Kit (QIAGEN, Hilden, Germany), following the manufacturer’s instructions. The RNA was eluted using 50 μL of elution buffer, aliquoted, and promptly stored at −80 °C until further use. Furthermore, the total RNA from the mammalian cells (HEK-293T, Vero E6, and Huh7) and mosquito cells (C6/36) was extracted using the RNeasy^®^ Mini Kit (QIAGEN, Hilden, Germany), following the manufacturer’s instructions, to be utilised as the negative controls. HEK-293T, Vero E6, and Huh7 cells were purchased from ATCC^®^, while a C6/36 cell line was obtained from the Institute of Tropical Medicine (Nagasaki University, Nagasaki, Japan), where it was initially established [28]. 

### 2.4. RT-PCR Assay

The DENV RNA was reverse-transcribed into cDNA using the ReverTra Ace kit (Toyobo, Osaka, Japan). The total volume of 20 μL of the reaction mixture consisted of 4 μL of 5X RT Buffer, 2 μL of a dNTP mixture (10 mM), 0.5 μL of an RNase inhibitor (10 U/μL), 1 μL of random primers (25 pmol/μL), 1 μL of ReverTraAce (100 U/μL), and a final amount of approximately 500 ng of extracted RNA. Before adding the RNA samples to the mixture, they were denatured at 65 °C for five min and immediately incubated on ice for one min. The reverse transcription reaction consisted of three steps: (1) annealing at 30 °C for 10 min, (2) an enzymatic reaction at 42 °C for 30 min, and (3) denaturation at 99 °C for 5 min. Following the reaction, the cDNA concentration and quality were measured with the NanoDrop^®^ ND-1000 spectrophotometer (Thermo Fisher Scientific, Wilmington, DE, USA). 

The PCR amplification was independently performed with KOD One^TM^ (Toyobo, Osaka, Japan), using the newly designed set of primers. The total volume of 50 μL of the reaction mixture consisted of 25 μL of KOD One PCR Master Mix (2×), 1.5 μL of each primer (10 mM), and a final amount of approximately 50 ng of cDNA. The amplification consisted of 30 cycles of denaturation at 98 °C, annealing at 55 °C, and elongation at 68 °C, all for 10 s. 

The PCR products were loaded onto a 1% agarose gel and subjected to electrophoresis. The results were visualised under an LED light. The DNA from the mammalian (HEK-293T, Vero E6, and Huh7) and mosquito (C6/36) cells were included in the PCR to determine the specificity of our primers. Moreover, cDNA from other flaviviruses, such as Japanese encephalitis virus (JEV), yellow fever virus (YFV), and Zika virus (ZIKV), were included. 

### 2.5. Sequencing

Positive PCR bands were excised from the gel and purified using the Wizard SV Gel kit from Promega (Madison, WI, USA) to assess the sequencing primers, following the manufacturer’s protocol. This DNA was used as the template for sequencing the PCR using the newly designed primers and the BigDye™ Terminator v3.1 Cycle Sequencing Kit (Thermo Fischer Scientific, Austin, TX, USA). The total volume of 10 μL of the reaction mixture consisted of 1 μL of BigDye™ Terminator 3.1 Ready Reaction Mix, 2 μL of BigDye™ Terminator v1.1 & v3.1 5X Sequencing, 1 μL of primer (3.2 μM), and a final amount of approximately 300 ng of cDNA. The reaction consisted of an initial denaturation at 96 °C for 1 min, followed by 25 cycles of 96 °C for 10 s, 50 °C for 5 s, and 60 °C for 1 min. 

Agencourt CleanSEQ (Beckman Coulter, Tokyo, Japan) was used according to the manufacturer’s instructions to remove the unincorporated dyes, nucleotides, salts, and contaminants from the PCR products. The samples were sequenced using a 3500 Genetic Analyser (Applied Biosystems, Tokyo, Japan). The results were analysed using Phred (Ver. 0.020425) and Phrap software (Ver. 0.990319) [29,30] to generate contigs for each sample.

### 2.6. In Silico PCR

Representative DENV sequences from the Americas were downloaded from the NCBI for Biotechnology Information and aligned using MEGA (v11.0.11) [31]. From the alignment of these sequences, a consensus sequence was obtained for each serotype, which was used to test our primers based on an in silico analysis using AmplifX2 (v2.1.1).

### 2.7. Viruses

The DENV strains from different serotypes isolated from the Philippines, Myanmar, Vietnam, Nepal, Sri Lanka, and Malaysia, along with isolates of JEV, YFV, and ZIKV, were propagated either on VeroE6 or C6/36 cells in Minimum Essential Medium (MEM). The supernatant was collected 3–7 days after infection. The virus titer was determined through a Focus assay, and the viruses were stored at −80 °C. Detailed information on the viral strains is presented in Table 1.

### 2.8. Clinical Specimens

In total, 29 human serum samples were used to evaluate the primer efficacy; 27 samples were acquired from the Vung Tau General Hospital, Vietnam. These samples consisted of serum specimens from patients in the acute phase of infection who were confirmed positive for DENV-1, DENV-2, and DENV-4 through PCR testing. One serum sample from a DENV-3-positive patient came from the Dhading Hospital, Eastern Nepal, and one sample was collected from a healthy individual affiliated with Nagasaki University.

## 3. Results

### 3.1. Primer Design

Our assay targeted the E gene of each DENV serotype. It consisted of two primers to amplify the entire region situated at the flanking sites of the gene and four primers to produce overlapping fragments for the Sanger sequencing. The resulting RT-PCR system obtained and sequenced the entire E gene. The nucleotide sequences and genomic positions of all primers and probes are listed in Table 2. The expected band sizes after amplification were 1740 bp for DENV-1, 1859 bp for DENV-2, 2335 bp for DENV-3, and 1747 bp for DENV-4. 

### 3.2. Viral Sample Detection and Specificity Assay

**Figure 2:**
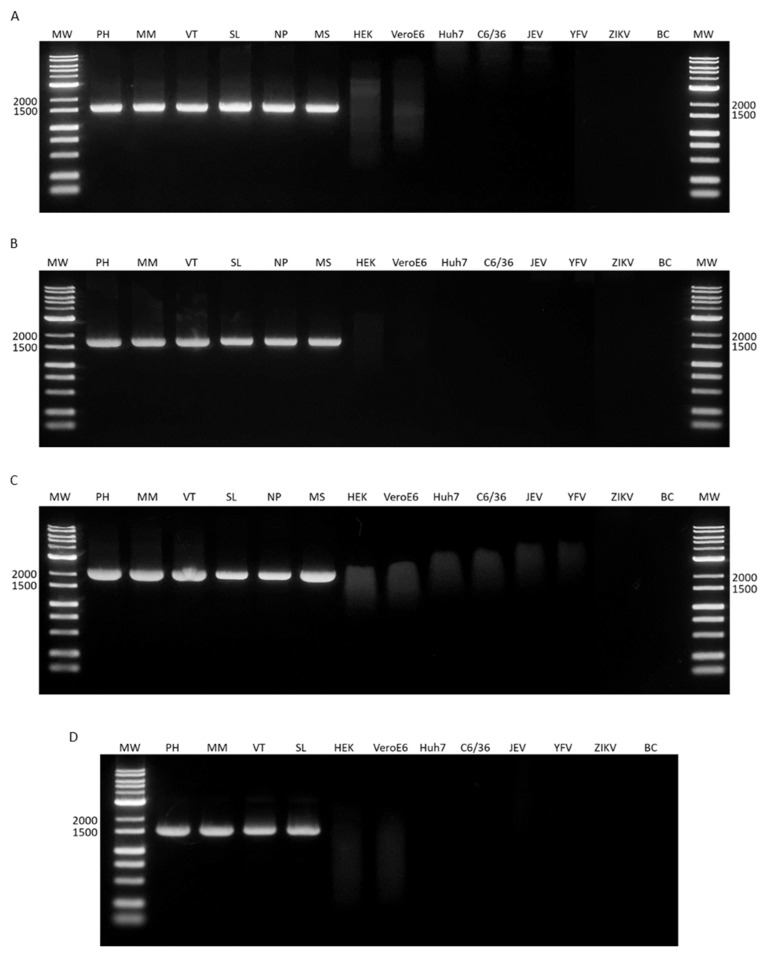
Specificity of primers when amplifying the full-length envelope gene of DENV isolates from the Philippines, Myanmar, Vietnam, Sri Lanka, Nepal, and Malaysia. The expected band sizes after amplification were (**A**) 1740 bp for the DENV-1 primer set, (**B**) 1859 bp for DENV-2, (**C**) 2335 bp for DENV-3, and (**D**) 1747 bp for DENV-4. MW, molecular weight marker; PH, the Philippines; MM, Myanmar; VT, Vietnam; SL, Sri Lanka; NP, Nepal; MS, Malaysia; HEK, HEK-293T cells; VeroE6, Vero E6 cells; Huh7, Huh7 cells; C6/36, C6/36 cells; JEV, Japanese encephalitis virus; YFV, yellow fever virus; ZIKV, Zika virus; and BC, blank control (water).

The wide detection range of the assay was evaluated using samples from the Philippines, Myanmar, Nepal, Sri Lanka, Malaysia, and Vietnam. Sets for each serotype were able to amplify the entire E gene, and DNA products of the expected sizes were obtained for all samples (Figure 2). The DNA from the mammalian (HEK-293T, Vero E6, and Huh7) and mosquito (C6/36) cells were used as a negative control to determine the assay’s specificity. The cDNA from other flaviviruses (JEV, YFV, and ZIKV) were also included. The DNA from uninfected cells and related flaviviruses was undetected (Figure 2).

### 3.3. Clinical Sample Detection

**Figure 3:**
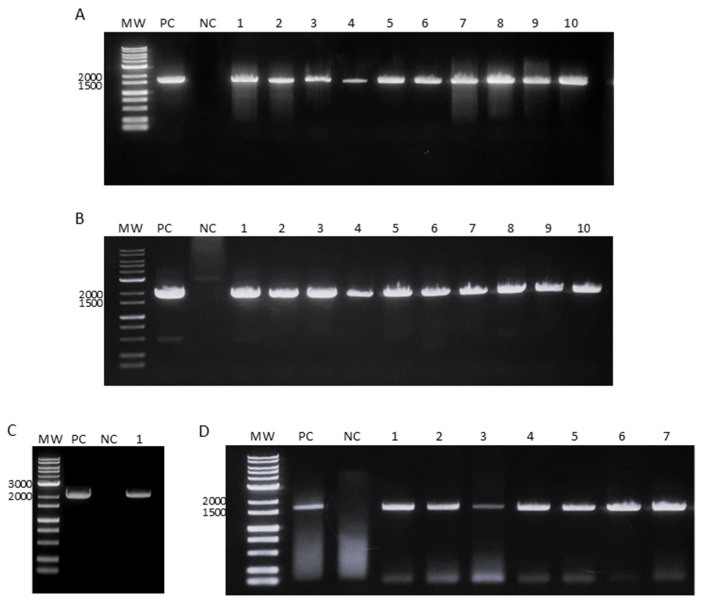
Amplification of the dengue virus (DENV) envelope gene from patient serum samples and healthy individuals (negative controls). (**A**) DENV-1, (**B**) DENV-2, (**C**) DENV-3, and (**D**) DENV-4. MW, molecular weight marker; PC, positive control; NC, serum from healthy individual (negative control); and 1–10, serum samples.

The cDNA from the serum samples derived from DENV-infected patients was tested using our assay to ensure that the assay was not negatively affected by components in the human samples. A sample from a healthy individual was used as the negative control. The results indicated that the assay was unaffected by human serum components and that our primers could efficiently amplify the DENV genome. In contrast, the negative clinical sample did not show amplification (Figure 3). 

### 3.4. Sensitivity Analysis

**Figure 4:**
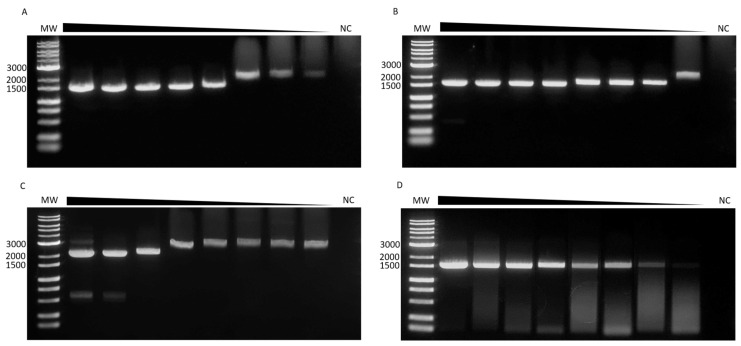
The detection limit of the primer sets used to amplify the envelope gene for each serotype. Ten-fold serial dilutions of DENV cDNA were prepared and tested in the PCR. (**A**) DENV-1, (**B**) DENV-2, (**C**) DENV-3, and (**D**) DENV-4. MW, molecular weight marker; and NC, negative control.

The detection limit of our primer sets was determined through 10-fold serial dilutions of cDNA from isolated viruses, with an initial concentration of 1 × 10^8^ DNA copies/μL. Our primers detected all the prepared dilutions, including those with the lowest concentrations (Figure 4). Notably, larger bands were observed at different DENV-1, DENV-2, and DENV-3 dilutions. However, it was possible to sequence the entire E gene after purifying the DNA from these bands and sequencing. 

Because we could not access samples from other parts of the world, we conducted an in silico PCR using samples from the Americas. Table S1 of the Supplementary Information presents detailed information on the strains used. We generated consensus sequences for each serotype. Although our primers did not exactly match these consensus sequences, they were successfully annealed and amplified in an in silico assay using Amplifx (Figure S1).

### 3.5. Sanger Sequencing

All the samples were sequenced using a forward primer for amplification and four other primers. We obtained five overlapping segments aligned using MAFFT for each sample, resulting in a complete E gene sequence. 

## 4. Discussion

The spread of DENV is becoming a major threat to public health worldwide. There is no specific antiviral treatment for dengue, so early detection is necessary for supportive management. Diagnosis is important to monitor outbreaks and implement appropriate control measures. Various diagnostic methods are available, including anti-DENV antibodies, nonstructural protein 1 (NS1) antigens, and DENV-specific nucleic acid detection. Owing to its specificity and ability to differentiate serotypes, RT-PCR has become the gold standard for dengue fever surveillance [32]. 

In this study, we present a simple, convenient, and standardised RT-PCR technique for amplifying and sequencing the full-length E gene of DENV from a wide range of geographical locations in Asia. Studies have shown that each DENV serotype comprises multiple lineages that are similar within the same region but different across different geographic regions. These lineages, known as genotypes, are typically found in specific regions of the world and exhibit differences of approximately 3% at the amino acid level and 6% at the nucleotide level [33]. The samples tested in this study comprised the most representative Asian genotypes for each serotype, all efficiently amplified. No cross-reactivity was detected between the type-specific primers, other flaviviruses, serum samples, and mammalian or mosquito DNA.

The E gene was sequenced using our new serotype-specific primers. Overall, the sequence quality was good, allowing us to cover the entire gene. However, typical Sanger sequencing often yields low-quality sequences within the first 15–40 bp due to primer binding [34]. Consequently, in some cases, we could not sequence the beginning of the gene because of the proximity of the DENV-1 and DENV-2 forward primers. This proximity leads to problems with base calling in the sequencing trace. Nevertheless, by meticulously repeating the purification step with Agencourt CleanSEQ and ensuring the thorough removal of all beads, we successfully obtained a sequence that covered the initial segment of the gene.

During the sensitivity testing of the primers, we observed PCR products that were larger than expected for certain DENV-1, DENV-2, and DENV-3 dilutions. However, we successfully sequenced the entire E gene using the PCR products. The sequencing of the DENV-2 products revealed an abrupt sequence termination at the 3′ end, where it was expected to end. As our assay lacks a reverse primer for the 5′ end of the E-coding region, we could not confirm this observation; however, we presume that the nonspecific fragment amplified is located towards this end. When sequencing the larger-than-expected products from DENV-1 and DENV-3, the signal of the sequence obtained from the primer covering the 3′ end slightly decreased towards the end but still covered part of the expected NS1 sequence; as such, the nonspecific fragment amplified could be located towards either end. We cannot explain the appearance of these nonspecific fragments; however, it is important to note that this did not affect the E sequencing in any way.

Currently, highly specific and sensitive RT-PCR methods are available for DENV diagnosis. These include the standard RT-PCR [35] and multiplex RT-PCR, which can detect and differentiate serotypes [36]. Other innovative methods include the use of a multiplexed PCR chip platform with an ion-selective membrane sensor [37] or a plasmonic colorimetric RT-PCR strategy [38]. The latest diagnostic methods have proven extremely sensitive and can even detect around 100 copies/μL of viral nucleic acid. However, these methods only amplify a small viral genome region to achieve this sensitivity and, therefore, cannot be used for phylogenetic analysis. 

Whole genome viral sequences are the most suitable options for obtaining accurate phylogenetic trees and enhancing epidemiological insights. Nonetheless, owing to the high cost and complexity, the analysis of specific genomic regions is the preferred approach. This method has been proven to accurately recapitulate the phylogeny of DENV compared with a whole genome sequence analysis [39]. 

Viral proteins comprise a combination of conserved and highly variable sections. The conserved sections contain amino acid residues crucial for the structure and function of the virus. Because these regions are vital for the survival of the virus, mutations are not tolerated. However, numerous mutations are tolerated in some regions, allowing the virus to evade the immune system through antigenic diversity [39,40]. The regions with the most mutations and the highest proportion of nonsynonymous mutations in DENV are situated within the E gene, NS2A, and the catalytic region of the NS5 RNA polymerase [39]. Owing to this significant mutation rate, the gene’s function in the viral entry into the host cell, and its role as the target of neutralising and enhancing antibodies, the E gene is most commonly used for a phylogenetic analysis. 

Several groups have previously reported the development of primer sets for PCR to amplify the E gene of one or multiple DENV serotypes and their subsequent sequencing [20,21,41,42,43]. However, laboratories use “in-house” methods, and none of these reports specify the methodology used to design the primers or the sequences used. Our report differs from these previous reports in that we designed our primers using a globally representative set of sequences for each serotype and validated them using various samples to verify their functionality. To the best of our knowledge, the only report that used a similar approach is from the CDC [22].

Genetic analysis of DENV is crucial for determining the molecular epidemiology of the virus, monitoring its introduction and possible routes of viral entry, and investigating the dispersion of viruses and the patterns of their evolution [17,18]. Therefore, we employed our methodology to investigate the 2022 DENV epidemic in Vietnam. Using our primers, we successfully amplified the E gene from 70 clinical samples, sequenced them, and conducted a phylogenetic analysis to explore the molecular epidemiology of the outbreak. Our approach enabled the sequencing of the DENV-1, DENV-2, and DENV-4 strains, identifying a new lineage of the DENV-2 cosmopolitan genotype in Vietnam, which was implicated in the outbreak [44]. Currently, we are applying the same methodology to examine the molecular epidemiology of DENV outbreaks in Nepal (comprising DENV-1, DENV-2, and DENV-3 strains) and Myanmar (encompassing strains from all serotypes). According to the in silico PCR results, although our primers did not precisely match the consensus sequence obtained for each serotype, they functioned effectively. This suggests that the same primers could be useful for studying DENV epidemics in the Americas, which also experiences virus hyperendemicity and is frequently affected by large outbreaks [45]. However, in silico matching may not accurately predict primer binding efficiency. While acknowledging that our primers may not yield successful results in American samples, it is noteworthy that a complementary technique utilising the primers developed by the CDC [22] provides the standardised tools for both regions most burdened by DENV.

In conclusion, the new set of degenerate serotype-specific primers presented here proved efficient for amplifying and sequencing the full-length E gene of DENV. Our method can detect a broad range of DENV strains effectively and is simple, cost-effective, and accessible to laboratories in dengue-endemic regions. Collectively, our methodology is a useful tool for epidemiological and evolutionary analyses in Asian countries constantly affected by dengue epidemics. 

## Figures and Tables

**Table 1:** Viruses used to determine the sensitivity of the RT-PCR.

Location	Serotype	Genotype	Strain
Philippines	DENV-1	IV	99St12A
	DENV-2	Asian II	00St22A
	DENV-3	I	SLMC50
	DENV-4	I	SLMC318
Myanmar	DENV-1	I	CH-156/2018
	DENV-2	Asian I	CH-348/2018
	DENV-3	I	CH-165/2018
	DENV-4	I	CH-120/2018
Vietnam	DENV-1	I	VT22.3473
	DENV-2	Cosmopolitan	VT22.3673
	DENV-3	I	01-TN-426
	DENV-4	I	VT22.3125
Sri Lanka	DENV-1	I	P-185/2019
	DENV-2	Cosmopolitan	N-36/2017
	DENV-3	III	N-189/2017
	DENV-4	I	N-92/2017
Malaysia	DENV-1	I	Mys-2/2019
	DENV-2	Cosmopolitan	Mys-12/2019
	DENV-3	III	Mys-66/2019
Nepal	DENV-1	V	Nepal-10726/2022
	DENV-2	Cosmopolitan	Nepal-14726/2022
	DENV-3	III	Nepal-10973/2022
Japan	JEV	III (OH0566)	
Ghana	YFV	17D vaccine strain	
Puerto Rico	ZIKV	Asian (PRVABC59)	

**Table 2:** Oligonucleotide primers used to amplify and sequence the E gene of DENV.

Primer ID	Sequence	Location ^1^
* DENV1EF	CACAYRCYATAGGRACATCCAT	858–879
DENV1Eseq1	ACGTGYGYYAARTTYRAGTGTGT	1277–1299
DENV1Eseq2	ACRTTYAAGACAGCYCATGCAAA	1649–1671
DENV1Eseq3	ARCAGAYGCRCCATGCAAGAT	1918–1938
DENV1Eseq4	GAGRYACHGCATGGGACTT	2181–2199
* DENV1ER	CTGATCGWATTCCACACACAC	2577–2597
* DENV2EF	ATCYTGGCATACACCATAGG	853–872
DENV2Eseq1	AGAGGATGGGGAAATGGATG	1231–1250
DENV2Eseq2	GAARTYAARRTAACACCACAGAG	1417–1439
DENV2Eseq3	RGCYACRGAAATCCARATGTC	1734–1754
DENV2Eseq4	AYATAGAAGCMGAACCYCCATT	2033–2054
* DENV2ER	ATRATWCCTTTRATGTCTCCTGTCAT	2686–2711
* DENV3EF	GCTGAAGGAGCTTGGAGAC	773–791
DENV3Eseq1	ARCCYACGYTRGAYATAGAGCT	1047–1068
DENV3Eseq2	TCATYACAGTKCACACAGGAGA	1353–1374
DENV3Eseq3	CRCAAGAGGGAGCAATGCA	1692–1710
DENV3Eseq4	RGGTGCAAGGCGCATG	2149–2164
* DENV3ER	ARAGWGTGTGTGATTTTGGCC	3087–3107
* DENV4EF	GARACRTGGATGTCATCGGA	762–781
DENV4Eseq1	CAAGATGTCCAACRCAAGGAGA	1153–1174
DENV4Eseq2	ATAACYCCYAGGTCACCATC	1428–1447
DENV4Eseq3	GGGAATGTCATACACKATGTG	1823–1843
DENV4Eseq4	GAYTTTGGYTCHGTTGGTGGA	2199–2219
* DENV4ER	TCCARGTGTGCACRTTGTC	2490–2508

^1^ The location of the primers are based on the reference sequence of each serotype. * The primers used to amplify the full-length E gene.

## Data Availability

The original contributions presented in the study are included in the article/Supplementary Material, further inquiries can be directed to the corresponding author.

## Supplementary Materials

The following supporting information can be downloaded from https://www.mdpi.com/article/10.3390/microorganisms12061092/s1. Figure S1: American DENV consensus sequences for each serotype and their alignment with the new amplifying primers. Table S1: Sequences used for In-Silico PCR of DENV isolates from the Americas.
