# Supplementary figures and images for: The Development of New Primer Sets for the Amplification and Sequencing of the Envelope Gene of All Dengue Virus Serotypes
Source: Microorganisms. 2024 May 28;12(6):1092. doi: 10.3390/microorganisms12061092 (PMC11205395; doi:10.3390/microorganisms12061092)

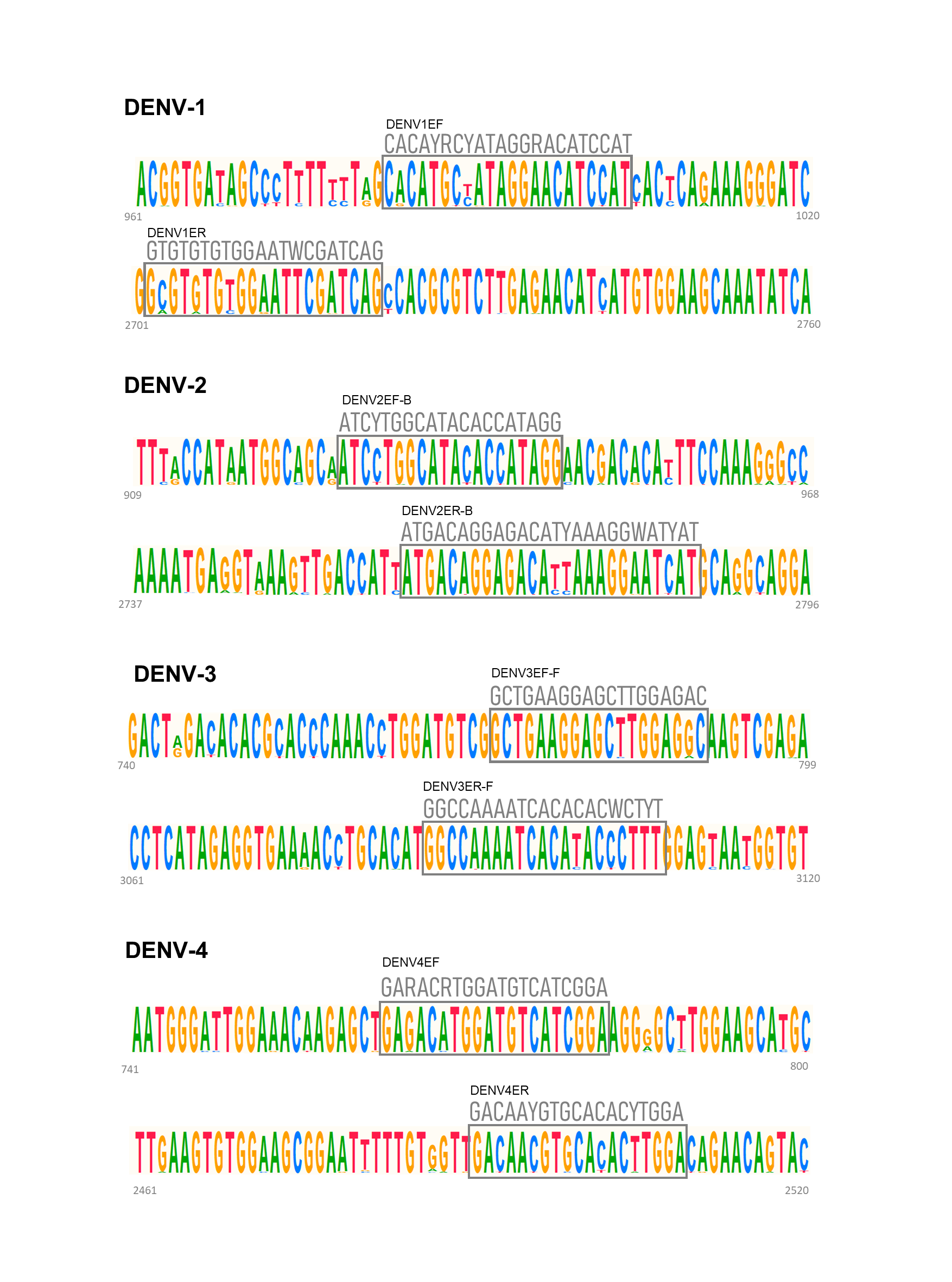

Supplement: Supplementary file 1 [file microorganisms-12-01092-s001.zip › Figure S1.tif]
